# Impacts of Environmental Changes on Well-Being in Indigenous Communities in Eastern Canada

**DOI:** 10.3390/ijerph17020637

**Published:** 2020-01-19

**Authors:** Laura Fuentes, Hugo Asselin, Annie Claude Bélisle, Oscar Labra

**Affiliations:** 1 École d’études Autochtones, Université du Québec en Abitibi-Témiscamingue, Rouyn-Noranda, QC J9X 5E4, Canada; marcela.laura406@gmail.com; 2 Institut de Recherche sur les Forêts, Université du Québec en Abitibi-Témiscamingue, Rouyn-Noranda, QC J9X 5E4, Canada; annieclaude.belisle@uqat.ca; 3 Département des Sciences du Développement Humain et Social, Université du Québec en Abitibi-Témiscamingue, Rouyn-Noranda, QC J9X 5E4, Canada; oscar.labra@uqat.ca

**Keywords:** Aboriginal people, wellness, environmental distress

## Abstract

Climate change and natural resource exploitation can affect Indigenous people’s well-being by reducing access to ecosystem services, in turn impeding transmission of traditional knowledge and causing mental health problems. We used a questionnaire based on the Environmental Distress Scale (EDS) and the Connor–Davidson Resilience Scale (CD-RISC-10) to examine the impacts of environmental changes on 251 members of four Indigenous communities in the eastern Canadian boreal forest. We also considered the potential mitigating effects of sociodemographic characteristics (i.e., age, gender, parenthood, and time spent on the land) and protective factors (i.e., health, quality of life, resilience, life on the land, life in the community, and support from family and friends). Using linear regression, model selection, and multi-model inference, we show that the felt impacts of environmental changes increased with age but were lower for participants with higher quality of life. The effect of resilience was opposite to expectations: more resilient participants felt more impacts. This could be because less resilient individuals ceased to go on the land when environmental changes exceeded a given threshold; thus, only the most resilient participants could testify to the impacts of acute changes. Further research will be needed to test this hypothesis.

## 1. Introduction

Climate change, added to an ever-increasing pressure to exploit natural resources, causes environmental changes that impact public health [1,2,3,4]. Indigenous peoples live in close connection with the land and are thus more directly affected by environmental changes [5,6,7]. Hence, environmental distress is experienced in Indigenous communities as a result of reduced well-being due to the lower access to ecosystem services, hindered transmission of traditional knowledge, and altered lifestyles [6,8,9,10,11].

Reading [12] used the metaphor of a tree to explore the social determinants of Indigenous health. The tree crown represents the proximal determinants such as age, gender, the physical environment, income, and social status. The tree roots represent distal (or structural) determinants, i.e., the historical, political, ideological, economical, and social foundations from which all other determinants evolve. The tree trunk represents intermediate determinants that facilitate or hinder health through connexions between proximal and distal determinants; they include social support, access to services, and relationship with the land.

Among the proximal determinants of health, different sociodemographic characteristics might influence how environmental changes affect Indigenous well-being. The more time someone spends on the land, the more likely he/she is to be affected by environmental changes. In addition, women and men use the land differently and this could be reflected in how they are affected by environmental changes [13,14,15,16,17]. Similarly, youth, adults, and elders do not use the land in the same way and could be affected differently by environmental changes [18,19,20,21,22]. Having children or not also results in different land use patterns and possible impacts of environmental changes.

The impacts of environmental changes on Indigenous well-being can also be mitigated by proximal and intermediate determinants of health acting as protective factors. More resilient individuals have a higher capacity to bounce back after a disturbance and could thus be expected to be less affected by environmental changes [10,23]. Higher quality of life and better health conditions are also expected to reduce the impacts of environmental changes [19,24]. Support from family and friends and good relations with others within communities or on the land could also mitigate the impacts of environmental changes [25,26].

Our main objective was to examine how environmental changes affect Indigenous well-being in the boreal forest of eastern Canada, taking into account sociodemographic characteristics and protective factors. We considered the following hypotheses:

**Hypothesis 1** **(H1).** Environmental distress increases when environmental changes increase.

**Hypothesis 2** **(H2).** The relationship between environmental distress and environmental changes varies according to sociodemographic characteristics (i.e., age, gender, time spent on the land and parenthood).

**Hypothesis 3** **(H3).** Environmental distress resulting from environmental changes is reduced by protective factors (i.e., resilience, health, quality of life, support from family and friends, life on the land, and life in the community).

We found that the more environmental changes were perceived, the more impacts were felt. Older participants felt more impacts from environmental changes, but no other sociodemographic characteristic had a significant effect. Participants with higher quality of life felt less impacts, whereas more resilient participants unexpectedly felt more, maybe because less resilient persons do not go on the land if environmental changes exceed a given threshold.

## 2. Materials and Methods

### 2.1. Study Area

The study area was in the eastern Canadian boreal forest, on the traditional territories of four communities belonging to three different Indigenous peoples: the Cree community of Ouje-Bougoumou (820 members), the Atikamekw community of Opitciwan (2697 members), and the Anishnaabeg communities of Pikogan (996 members) and Wahgoshig (303 members). These communities experience various degrees of environmental changes, both in number and intensity of stressors (e.g., climate change, forestry, mining, hydropower development).

The three Indigenous peoples to which the participating communities belong are part of the Algonquian language family, share several cultural traits, and all used to follow a nomadic lifestyle based on hunting, trapping, fishing, and gathering before forced settlement in the 20th century [27,28]. Education levels are generally low, and the unemployment rate is high. The main sources of employment are public services, administration, education, health, and development of community infrastructure.

### 2.2. Data Collection

We designed a questionnaire based on the Environmental Distress Scale (EDS) and on the 10–item Connor–Davidson Resilience Scale (CD-RISC 10) (see Supplementary Materials). The EDS was developed to evaluate the impacts of environmental changes on human distress in rural and Indigenous communities in Australia [29]. It was also used in a study with the Rigolet Inuit community in Labrador, eastern Canada [30]. We used two sections of the EDS to measure people’s observations of environmental changes (15 items) and felt effects of environmental changes (22 items). Items were measured on a 5 point Likert scale.

We also measured participants’ sociodemographic characteristics (4 items) to determine if they influenced the link between perceived environmental changes and felt impacts: gender (man or woman); age group (18–35 or >35 years old); parenthood (having children or not); and time spent on the land (never, a few times a year, a few times a month, a few times a week, always). We also measured protective factors with slightly modified items of the EDS (all measured on a 5 point Likert scale): life in the community (14 items); life on the land (7 items); support from family and friends (5 items); quality of life (1 item), and health (1 item). We measured resilience with the 10–item (each measured on a 4 point Likert scale) Connor–Davidson Resilience Scale [31] which was validated for use with different ethnic groups around the world including Indigenous peoples [31,32,33].

### 2.3. Ethical Considerations

We respected the principles of ethical research with Indigenous peoples [34]. Each community provided approval and each individual participant completed a consent form [35]. We obtained an ethics certificate from the Ethics Review Board of Université du Québec en Abitibi-Témiscamingue in April 2016 (#2016-04-Asselin). The names of the participants were not collected to ensure confidentiality. We conducted the survey between June and November 2016. All participants were 18 years of age or older. The time to complete the survey averaged approximately 30–45 min per person. Liaisons within each community helped recruiting participants who were contacted in the streets, in their homes or offices, and at public gatherings.

### 2.4. Analyses

We used three series of linear regressions and model selection based on the Akaike information criterion (AICc) to assess the relationship between perceived frequency of environmental changes and felt impacts of changes (H1) as well as the effects of sociodemographic characteristics (H2) and protective factors (H3) on this relationship. We conducted statistical analyses with version 3.4.4 of the R software using the base package and the AICc modavg package [36]. We considered parameters with a confidence interval excluding zero to have significant effects. We verified the application conditions for all regression series with a visual examination of the validation plots for the general models (with all variables). We checked outliers for possible errors during data entry. We performed the analyses with and without outliers and, as the results were similar, we kept the outliers. We considered models with a delta AICc ≤ 2.

We verified the reliability and internal consistency of the EDS sections (0.48–0.84) and CD-RISC 10 (0.85) with Cronbach’s alpha. Except for life in the community (0.48), all other variables had Cronbach’s alpha values higher than 0.70 which was deemed acceptable. We tested the effect of the perceived frequency of environmental changes on felt impacts (H1) using a linear regression. We subsequently analyzed the effects of sociodemographic characteristics (i.e., gender, age, time spent on the land, and parenthood) on the relationship between perceived frequency of environmental changes and felt impacts (H2). We used model selection to compare the contributions of sociodemographic characteristics and to identify the most parsimonious combination explaining environmental distress [37]. We ranked sociodemographic characteristics using model averaging. We calculated the weight of a variable by summing the weights of all models including it [37]. The most parsimonious model was then selected to test if protective factors (i.e., resilience, support from family and friends, life in the community, life on the land, health, and quality of life) reduced the impacts of perceived environmental changes (H3). We thus performed a second model selection with felt impacts as a response variable and perceived frequency of change, retained sociodemographic characteristics, and all possible combinations of protective factors as explanatory variables. We weighted the contributing factors using model averaging (as for H2).

## 3. Results

A total of 251 persons completed the survey (126 women, 125 men) (Table 1). Highly correlated variables were deleted (health and life on the land). Participants were initially assigned to one of three age groups: 18–35, 35–65, and ≥66 years old. However, only a few (13) seniors accepted to participate. Most mentioned they prefer interviews, as they can detail and contextualize their answers rather than answer closed-ended questions. Hence, age was reclassified into only two groups for analyses: 18–35 and ≥36 years old. The majority of participants were older than 35 years old (68.5%). Between 5% and 40% of the adults living in the communities took part in the survey. Most participants had children (77%). Time spent on the land was a few times a year/month/week for most participants (47%, 26%, and 11%, respectively) with some living full time on the land (14%) and only 2% never going.

### 3.1. Impacts of Environmental Changes

We tested the link between perceived frequency of environmental changes and felt impacts using linear regression (H1). The confidence interval of the coefficient for felt impacts excluded zero (Table 2), confirming the collinearity of this variable with perceived frequency of environmental changes. Thus, H1 was verified: the more someone perceived environmental changes, the more impacts he/she felt.

### 3.2. Effects of Sociodemographic Characteristics on Felt Impacts of Environmental Changes

We used model selection based on AICc to assess the effects of sociodemographic characteristics on the relationship between perceived frequency of environmental changes and felt impacts (H2). A total of 16 models were tested, of which four had a delta AICc ≤ 2 and were thus considered (Table 3). All retained models included age. Model 12 was the most parsimonious and only included age (confidence interval excluding zero; Table 4); it was thus selected for further analyses (see below). We summed cumulative AICc weights and age had the highest weight (age = 0.75; time spent on the land = 0.42; parenthood = 0.37; gender = 0.35).

Felt impacts of environmental changes varied according to age, and, thus, H2 was partly verified. Older participants (≥36 years old) felt more impacts than younger participants (18–35 years old) for the same frequency of environmental changes. However, all participants regardless of age group felt the same (highest) impacts for the highest frequency of environmental changes. None of the other sociodemographic characteristics affected felt impacts of environmental changes.

### 3.3. Role of Protective Factors in Mitigating Environmental Distress

We used model selection based on AICc to assess the effects of protective factors on the relationship between perceived frequency of environmental changes and felt impacts (H3). Of the 16 tested models, three had a delta AICc ≤ 2 and were thus considered (Table 5). All retained models included resilience and quality of life. Model 5 was the most parsimonious combination of variables and included resilience and quality of life (confidence interval excluded zero; Table 6). Resilience increased the felt impacts of environmental changes, whereas quality of life had the opposite effect. Resilience had the highest cumulative AICc weight (resilience = 1.00; quality of life = 0.83; life in the community = 0.76; support from family and friends = 0.38).

## 4. Discussion

### 4.1. Felt Impacts of Environmental Changes

Hypothesis 1 was confirmed: the more participants perceived environmental changes, the more impacts they felt. Previous research in Australia and Canada has shown that Indigenous people consider environmental changes as a hazard not only affecting the land but also their mental health [38,39]. With increasing environmental changes as a result of natural resource exploitation and climate change in Canada, environmental distress will likely increase in Indigenous populations [3,11,39,40,41].

### 4.2. Influence of Sociodemographic Characteristics on Felt Impacts of Environmental Changes

Hypothesis 2 was partly confirmed, as older participants (≥36 years old) felt more impacts than younger participants for a given level of environmental change, hence supporting the assertion that attachment to the land increases with age [42]. As older persons spent more time on the land than younger persons [8,43], they felt more impacts, although the effect of time spent on the land was not significant (see below). Some of the oldest participants might have felt more impacts because they had responsibilities on the land as suggested by previous work with Cree tallymen [27].

Because of the low participation of elderly people (≥66 years old), it was not possible to compare the answers of participants 36‒65 years old and older than 65 years old. Older participants had difficulty understanding the abstract concepts that the questions conveyed, and that were hard to translate into their native languages. Furthermore, they mentioned they felt more comfortable with open questions rather than multiple-choice questions, as they prefer to explain and contextualize their answers. Closed-ended questions therefore do not appear appropriate to work with older Indigenous people, and qualitative methods are better suited [44,45].

Gender did not significantly affect the impacts felt from perceived environmental changes. This could be explained by the fact that men and women share common values and cultural systems that influence their perception of environmental changes in a similar way [46,47].

Parenthood did not significantly affect the impacts felt from perceived environmental changes. Parenthood was expected to play a role, because the land is a privileged setting for cultural transmission as revealed, for example, by previous work with the Atikamekw people [8]. Maybe the lack of an effect of parenthood is due to the fact that Indigenous peoples in Canada tend to live in an extended family setting where everyone (parent or not) contributes to children’s education [48,49].

Time spent on the land did not significantly affect the impacts felt from perceived environmental changes. While it is possible that participants spending more time on the land were more exposed to environmental changes, the perceived frequency of environmental changes was controlled in the model. Moreover, attachment to the land might not be directly associated with the amount of time spent on the land. There is cultural transmission between persons spending more time on the land and those spending more time in the community [50,51], and social cohesion could mean that all community members share attachment to the land as well as distress associated with changes regardless of time spent on the land [52,53].

Other sociodemographic characteristics not tested here might affect the impacts felt from perceived environmental changes such as family composition, employment or education [1,54]. In addition, someone can feel distressed not only in face of environmental changes but also because of other situations with which he/she must cope [54].

### 4.3. Influence of Protective Factors in Mitigating the Impacts of Environmental Changes

Hypothesis 3 was partially confirmed, as resilience and quality of life significantly influenced the felt impacts of environmental changes. As expected, persons with a higher quality of life felt fewer impacts of environmental changes [39,54,55]. However, while resilience was also expected to reduce the felt impacts of environmental changes [56], the relationship was in the other direction: more resilient participants felt more impacts of environmental changes. As resilience was positively associated with felt impacts, but also with perceived frequency of environmental changes (data not shown), it could be that only the most resilient persons continue to go on the land when environmental changes are frequent, and thus they perceive more changes and feel more impacts. However, as they are resilient, they have the adaptive capacity to cope with changes [57]. Conversely, persons with low resilience might cease to go on the land when it is highly disturbed, as they are not able to handle so much change. More research is needed to test this assertion.

Support from family and friends did not have a protective effect on felt impacts of perceived environmental changes. In three of the four participating communities, the territory is divided into family hunting grounds, and thus family members are all exposed to the same level of environmental changes and likely experience similar distress. Previous studies have shown that support from family and friends is less efficient when distress is spread throughout the family [58].

Life in the community also did not have a protective effect on felt impacts of perceived environmental changes. The low internal consistency of this variable (Cronbach’s alpha = 0.48) could explain why its predictive power was low. Furthermore, while life in the community might turn people away from the traditional way of life [59], having a job can also provide the money needed to pursue traditional activities on the land [60]. Mobility among places increases adaptability to different environments and situations [10,61].

## 5. Conclusions

When Indigenous people perceive more environmental changes in the eastern Canadian boreal forest, they feel more impacts on their well-being. This is especially true for older individuals, for those who have a low quality of life, and likely for those that are less resilient. Environmental distress will continue to increase in the study area, as climate change will continue in the next decades and as the pressure to extract natural resources will continue to rise.

Limitations to this study could have influenced the results. First, some factors that could possibly affect people’s distress were not included such as education, employment, access to services, or family composition. Nevertheless, we likely took at least part of their effects into account by considering overarching variables such as quality of life, support from family and friends, and resilience. Second, due to the low participation from the oldest age group (>66 years old), we had to use a combined age class (≥36 years old) which could have masked some of the variability. Indeed, older Indigenous people are more often responsible for family hunting grounds and have a deeper connection with the land. Yet, the significant difference between the level of impacts felt by the younger and older age groups was consistent with expectations.

Two possible solutions to reduce environmental distress are (1) to refuse resource development projects beyond a certain threshold of environmental change where felt impacts exceed the resilience capacity of the more vulnerable community members; and (2) to develop measures to increase protective factors, especially resilience and quality of life.

## Figures and Tables

**Table 1:** Number of participants from each community according to gender and age group.

Nation	Community	Gender	Age Group			Total
			18–35	36–65	>66	
Cree	Ouje-Bougoumou	men	13	25	2	74
		women	16	16	2	
Anishnaabeg	Wahgoshig	men	8	12	0	38
		women	11	6	1	
	Pikogan	men	5	20	5	66
		women	6	27	3	
Atikamekw	Opitciwan	men	9	26	0	73
		women	11	27	0	
Total			79	159	13	251

**Table 2:** Specifications of the linear regression of felt impacts as a function of perceived frequency of environmental changes (H1).

Hypothesis	Variable	Coefficients	Standard Error	Confidence Interval (95%)
**H1**	Perceived frequency of environmental changes	0.40	0.05	(0.25–0.46)
**Intercept**		61.10		(58.42–67.34)
**Adjusted *R*^2^**		0.16		

**Table 3:** Linear models determining the effects of sociodemographic characteristics on felt impacts of perceived environmental changes. Retained models are shown in bold.

Model	Variables	Delta AICc	AICc Weight	Cumulative Weight
**12**	**FeltImpacts ~ FreqChange + Age**	**0.00**	**0.19**	**0.19**
**10**	**FeltImpacts ~ FreqChange + Age + TimeLand**	**0.70**	**0.14**	**0.33**
**2**	**FeltImpacts ~ FreqChange + Gender + Age**	**1.10**	**0.11**	**0.44**
**11**	**FeltImpacts ~ FreqChange + Age + Parenthood**	**1.51**	**0.09**	**0.53**
9	FeltImpacts ~ FreqChange + Age + TimeLand + Parenthood	2.04	0.07	0.60
5	FeltImpacts ~ FreqChange + Gender + Age + TimeLand	2.12	0.07	0.67
6	FeltImpacts ~ FreqChange + Gender + Age + Parenthood	2.55	0.05	0.72
14	FeltImpacts ~ FreqChange + TimeLand + Parenthood	3.05	0.04	0.77
15	FeltImpacts ~ FreqChange + Parenthood	3.25	0.04	0.80
8	FeltImpacts ~ FreqChange + Gender + Age + TimeLand + Parenthood	3.44	0.03	0.84
13	FeltImpacts ~ FreqChange + TimeLand	3.44	0.03	0.87
0	FeltImpacts ~ FreqChange	3.44	0.03	0.91
4	FeltImpacts ~ FreqChange + Gender + Parenthood	3.95	0.03	0.93
1	FeltImpacts ~ FreqChange + Gender	4.21	0.02	0.96
7	FeltImpacts ~ FreqChange + Gender + TimeLand + Parenthood	4.25	0.02	0.98
3	FeltImpacts ~ FreqChange + Gender + TimeLand	4.67	0.02	1.00

**Table 4:** Specifications for the regression of felt impacts as a function of frequency of environmental changes and age (H2, Model 12).

Hypothesis	Variables	Coefficients	Standard Error	Confidence Intervals (95%)
**H2**	Frequency Age (≥36)	0.34 2.90	0.05 1.22	(0.24–0.45) (0.46–5.30)
**Intercept**		61.14		(56.70–65.60)
**Adjusted *R*^2^**		0.20		

**Table 5:** Linear models determining the effects of protective factors on felt impacts of perceived environmental changes. Retained models are shown in bold.

Model	Variables	Delta AICc	AICc Weight	Cumulative Weight
**5**	**FeltImpacts ~ FreqChange + Age + Resil + Qual + Commun**	**0.00**	**0.37**	**0.37**
**8**	**FeltImpacts ~ FreqChange + Age + Resil + Qual + Commun + Supp**	**0.85**	**0.24**	**0.61**
**2**	**FeltImpacts ~ FreqChange + Age + Resil + Qual**	**1.94**	**0.14**	**0.75**
3	FeltImpacts ~ FreqChange + Age + Resil + Comm	2.66	0.10	0.84
6	FeltImpacts ~ FreqChange + Age + Resil + Qual + Supp	3.00	0.08	0.93
7	FeltImpacts ~ FreqChange + Age + Resil + Comm + Supp	3.96	0.05	0.98
1	FeltImpacts ~ FreqChange + Age + Resil	6.38	0.02	0.99
4	FeltImpacts ~ FreqChange + Age + Resil + Supp	7.94	0.01	1.00
9	FeltImpacts ~ FreqChange + Age + Qual + Comm + Supp	15.36	0.00	1.00
11	FeltImpacts ~ FreqChange + Age + Qual + Supp	15.71	0.00	1.00
14	FeltImpacts ~ FreqChange + Age + Comm + Supp	16.57	0.00	1.00
12	FeltImpacts ~ FreqChange + Age + Qual	17.64	0.00	1.00
10	FeltImpacts ~ FreqChange + Age + Qual + Comm	17.78	0.00	1.00
13	FeltImpacts ~ FreqChange + Age + Comm	18.09	0.00	1.00
15	FeltImpacts ~ FreqChange + Age + Supp	18.18	0.00	1.00
0	FeltImpacts ~ FreqChange + Age	19.00	0.00	1.00

**Table 6:** Specifications for the regression of felt impacts as a function of frequency of environmental changes, age, resilience, and quality of life (H3, Model 5).

Hypothesis	Variables	Coefficients	Standard Error	Confidence Intervals (95%)
**H3**	Frequency	0.30	0.05	(0.20–0.40)
	Age (≥36)	2.83	1.20	(0.50–5.20)
	Resilience	0.44	0.10	(0.24–0.62)
	Quality of life	−1.36	0.63	(−2.60–−0.12)
	Life in the community	−0.06	0.03	(−0.11–−0.00)
**Intercept**		59.27		(51.80–66.80)
**Adjusted *R*^2^**		0.22		

## Supplementary Materials

The following are available online at https://www.mdpi.com/1660-4601/17/2/637/s1, Questionnaire.
