# Supplementary material for: Impacts of Environmental Changes on Well-Being in Indigenous Communities in Eastern Canada
Source: Int J Environ Res Public Health. 2020 Jan 19;17(2):637. doi: 10.3390/ijerph17020637 (PMC7042811; doi:10.3390/ijerph17020637)
Supplement: Supplementary file 1 [file ijerph-17-00637-s001.pdf]

Supplementary material for:
Fuentes, L., Asselin, H., Bélisle, A. C. and Labra, O. 2020. Impacts of environmental changes on well-being in Indigenous communities in eastern Canada. *International Journal of Environmental Research and Public Health*.

Questionnaire

Sociodemographic characteristics

For each statement, indicate the one that applies to your situation

1. □ man □ woman
2. Age : □ 18–35 □ 36–65 □ 66 and over
3. Children : □ yes □ no
4. How often do you go to your family trapline in a typical year?

   □ never □ few times a year □ few times a month □ few times a week □ always

Quality of life and health

1. Generally, would you say your quality of life is:

   □ excellent □ really good □ good □ fair □ bad

2. Generally, would you say your health is:

   □ excellent □ really good □ good □ fair □ bad

   * As this variable was correlated with quality of life, it was not used in the analyses.

Supplementary material for:
Fuentes, L., Asselin, H., Bélisle, A. C. and Labra, O. 2020. Impacts of environmental changes on well-being in Indigenous communities in eastern Canada. *International Journal of Environmental Research and Public Health*.

Life in the community

For each of the following statements about life in your community, please indicate if you strongly agree (5), agree (4), neither agree nor disagree (3), disagree (2), strongly disagree (1).

	5	4	3	2	1
1. Generally, I am much attracted by the life in my community.					
2. Even if I had the opportunity to leave the community, I would still continue to live there.					
3. I feel like I belong to my community.					
4. I feel a sense of responsibility toward the members of my community.					
5. I appreciate the spirit of mutual help that prevails in my community.					
6. There are conflicts between different groups in my community.					
7. I would rather live somewhere else.					
8. My community is not a place to raise a family.					
9. People work together for the well-being of my community.					
10. Globally, I consider that there is strong dynamism in my community.					
11. The band council defends the interests of my community.					
12. I participate to events organized in my community.					
13. Governments invest sufficiently in my community.					
14. Companies invest sufficiently in my community.					

*The percentage of items with 4 or 5 as an answer was used in the analyses, taking into account reversed items.

Life on the land

For each of the following statements about life on your family trapline, please indicate if you strongly agree (5), agree (4), neither agree nor disagree (3), disagree (2), strongly disagree (1).

	5	4	3	2	1
1. I am proud of the heritage and history of my land.					
2. My identity is linked to my land.					
3. I get comfort and peace of mind from my land.					
4. I feel I know every rock, nook and cranny around my land.					
5. I feel a deep connection to my land.					
6. I feel I have a duty to maintain the land for future generations.					
7. Because of the changes to the land, I would leave if I could.					

*As this variable was correlated to life in the community, it was not used in the analyses.

Support from family and friends

For each of the following statements about the support you receive from people near you, please indicate how often you have access to such support when you need it: always (5), often (4), sometimes (3), rarely (2), never (1)

	5	4	3	2	1
1. A person who advises you concerning an important decision you must take.					
2. A person to whom you can confide and talk about personal and intimate things.					
3. A person to tell you that you have good ideas, that you are taking good decisions.					
4. A person with whom you can make activities to change your mind, have a good time.					
5. A person that can provide you with traditional food or other products from the land.					

*The percentage of items with 4 or 5 as an answer was used in the analyses.

Resilience (CD-RISC-10)

Please indicate how much you agree with the following statements as they apply to you over the last **month**. *If a particular situation has not occurred recently, answer according to how you think you would have felt. True nearly all the time (4), often true (3), sometimes true (2), rarely true (1), not true at all (0)*

	4	3	2	1	0
1. I am able to adapt when changes occur.					
2. I can deal with whatever comes my way.					
3. I try to see the humorous side of things when I am faced with problems					
4. Having to cope with stress can make me stronger.					
5. I tend to bounce back after illness, injury, or other hardships.					
6. I believe I can achieve my goals, even if there are obstacles.					
7. Under pressure, I stay focused and think clearly.					
8. I am not easily discouraged by failure.					
9. I think of myself as a strong person when dealing with life's challenges and difficulties.					
10. I am able to handle unpleasant or painful feelings like sadness, fear, and anger.					

*The sum of all answers was used in the analyses.

Frequency of environmental change

For each of the following statements about environmental changes, please indicate their frequency of occurrence on your family trapline: almost always (5), often (4), sometimes (3), rarely (2), never (1)

	5	4	3	2	1
1. Forest harvesting					
2. Mining exploration (claim lines, etc.)					
3. Mining exploitation (mine)					
4. Dams, reservoirs, hydroelectric facilities (plants, substations, transport lines)					
5. Wind turbines					
6. Roads (usable by car)					
7. Non-Aboriginal hunting camps					
8. Air pollution from industrial activities					
9. Noise from industrial activities					
10. Vibrations from industrial activities					
11. Soil pollution from industrial activities					
12. Water pollution from industrial activities					
13. Forest fires					
14. Insects outbreaks					
15. Unpredictable weather (temperature, rainfall, storms)					

*The sum of all answers was used in the analyses.

Felt impacts

For each of the following statements about possible impacts of environmental changes, please indicate if you strongly agree (5), agree (4), neither agree nor disagree (3), disagree (2), strongly disagree (1)

	5	4	3	2	1
1. Economic benefits of industrial development (e.g. jobs) are more important than any concerns I might have about the local environment.					
2. Economic benefits of industrial development are equitably shared between community members.					
3. I am unable to enjoy life as much as I would like because of local environmental changes.					
4. I feel positive about local environmental changes.					
5. Claims about sickness being caused by environmental pollution are exaggerated.					
6. My community is divided by disagreements about environmental issues.					
7. People I know have become physically ill because of pollution in the local environment.					
8. I am upset at the destruction of cultural sites due to industrial development.					
9. I am disturbed that decisions about development activity here do not give higher priority to long-term land use for future generations.					
10. My community receives its fair share of benefits from industrial development on the land.					

11. Industry funding of community projects is genuinely useful to my community.					
12. My ability to make a living has been negatively affected by environmental problems.					
13. I feel angry about degradation of my family trapline.					
14. I am worried about risks to human health from pollution.					
15. I am concerned that future generations will not be able to enjoy the natural environment.					
16. I am frustrated because I can't influence decisions about the development of the land.					
17. I am concerned environmental problems will cause illness to myself or my family.					
18. I am satisfied with the governments' efforts to monitor environmental impacts from industrial development.					
19. People in this area feel frustrated because the band council and community employees have limited power to influence environmental decisions.					
20. People I know have given up trying to preserve the environment because they feel powerless.					
21. The overall impact of industrial development on the land is depressing.					
22. People I know have become disillusioned trying to negotiate their rights in relation to the impact of industrial development.					

*The sum of all answers was used in the analyses, taking into account reversed items.
